# Advancing Precision Medicine: Recent Innovations in Gene Editing Technologies

**DOI:** 10.1002/advs.202410237

**Published:** 2025-03-02

**Authors:** Abhijith Koodamvetty, Saravanabhavan Thangavel

**Affiliations:** ^1^ Centre for Stem Cell Research (CSCR) A unit of InStem Bengaluru Christian Medical College campus Vellore Tamil Nadu 632002 India; ^2^ Manipal Academy of Higher Education Manipal Karnataka 576104 India

**Keywords:** CRISPR‐Cas9, gene editing, hematological disorders, prime editing

## Abstract

The advent of gene editing has significantly advanced the field of medicine, opening new frontiers in the treatment of genetic disorders, cancer, and infectious diseases. Gene editing technology remains a dynamic and promising area of research and development. Recent advancements in protein and RNA engineering within this field have addressed critical issues such as imprecise edits, poor editing efficiency, and off‐target effects. Advancements in delivery methods have allowed the achievement of therapeutic or even selection‐free gene editing efficiency with reduced toxicity in primary cells, thereby enhancing the safety and efficacy of gene manipulation. This progress paves the way for transformative changes in molecular biology, medicine, and other fields. This review provides a comprehensive overview of the advancements in gene editing techniques, focusing on prime editor proteins and their engineered variants. It also explores alternative systems that expand the toolkit for precise genomic modifications and highlights the potential of these innovations in treating hematological disorders, while also discussing the limitations and challenges that remain.

## Graphical abstract

This review article delves into the cutting‐edge advancements in gene editing technology, with a particular focus on prime editor proteins and their engineered variants. The authors provide a thorough examination of recent developments that have addressed key challenges in the field, including improved precision, enhanced editing efficiency, and reduced off‐target effects.

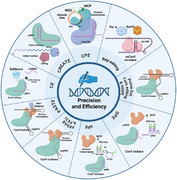

## 1 Introduction

Modifying pathogenic variations in the genome has been an aspiration since the discovery of genetic diseases. While initial efforts at gene manipulation using homologous recombination were of very low efficiency, zinc finger nuclease (ZFN) and transcription activator‐like effector nuclease (TALENs) have significantly improved gene editing efficiency.^[^ ^1^, ^2^ ^]^ The engineered zinc finger proteins or TALEs bind to specific DNA sequences and induce double‐strand breaks (DSBs) using Fok1 nuclease tethered to their DNA‐binding motifs, facilitating target gene alteration. Despite their effectiveness, these methods required labor‐intensive custom protein design for each target, making it challenging to test several targets or conduct high‐throughput screens.^[^ ^1^, ^2^, ^3^ ^]^ In addition, the effector, Fok1 nuclease, functions only as a dimer, thus necessitating the delivery of 2 zinc fingers/TALEs fused to Fok1 to bind to opposite DNA strands. The development of CRISPR‐Cas enzymes has addressed these challenges by using RNA instead of proteins for DNA targeting. The relative ease of designing and synthesizing single guide RNAs (sgRNAs) that base pairs with the target DNA sequences, coupled with the monomeric Cas9 as an effector nuclease to introduce DNA‐DSBs simplified the preparations for gene manipulation.^[^ ^4^, ^5^, ^6^ ^]^

The repair mechanisms such as non‐homologous end joining (NHEJ) and microhomology‐mediated end joining (MMEJ) can inadvertently introduce insertions and deletions (Indels), translocations, or other rearrangements at the DSB site, potentially disrupting the expression of genes.^[^ ^7^ ^]^ Another repair mechanism, homology‐directed repair (HDR), offers a more precise approach to DNA editing. HDR utilizes synthetic donor templates with homologous sequences to repair DSBs. This process allows for the accurate incorporation of specific nucleotide modifications or the insertion of transgenes flanked by homologous regions.^[^ ^8^ ^]^

The rapid advancement of CRISPR‐Cas9 technology in clinical applications and its promising therapeutic outcomes in various studies underscore the effectiveness of this system. The recent U.S. FDA approval of CASGEVY (exagamglogene autotemcel) for treating sickle cell disease (SCD) and transfusion‐dependent beta‐thalassemia represents a significant milestone as the first CRISPR‐based therapy to receive regulatory authorization. Additionally, several ongoing phase 1/2 clinical trials have yielded positive results, including in vivo gene editing approaches for conditions such as transthyretin amyloidosis (ATTR) and Leber congenital amaurosis. These developments collectively highlight the growing potential of CRISPR‐Cas9 as a powerful tool in the field of genetic medicine.^[^ ^9^, ^10^, ^11^ ^]^

The success of gene modifications depends heavily on the chosen repair pathway and the desired editing can be accompanied by undesired events. To harness more ubiquitous repair mechanisms, counter the undesired events, and broaden the gene‐editing repertoire, researchers have introduced new CRISPR‐associated gene editors, Base editing (BE) and Prime editing (PE),^[^ ^12^, ^13^ ^]^ which demonstrate the capability to precisely create nucleotide substitutions.^[^ ^14^, ^15^, ^16^ ^]^ PE, in particular, addresses some of the constraints associated with traditional CRISPR methods, mitigating bottlenecks that have hindered its therapeutic and biotechnological utility. In this context, we explored the fundamentals of PE and examined notable advanced variations designed to broaden the scope of precise gene editing.

## 2 Base Editing: The precursor to Prime Editing Technology

Base editing is the first next‐generation gene‐editing tool capable of precisely modifying nucleotides without requiring DNA DSBs. It employs Cas9 nickase fused with a DNA deaminase enzyme to create 2 main types of BEs, Cytosine Base Editors (CBEs) and Adenine Base Editors (ABEs).

CBEs: In cellular systems, cytidine deaminase catalyzes the irreversible hydrolytic deamination of cytidine and deoxycytidine, converting them to uridine and deoxyuridine, respectively. This process is crucial in the pyrimidine salvage pathway. When fused to Cas9 nickase, cytidine deaminase deaminates cytosine to uracil within the specific editing window generated by Cas9. The uracil glycosylase inhibitor (UGI), which is fused with cytosine deaminases, helps with the persistence of the newly created uracil by blocking uracil‐DNA N‐glycosylase (UNG2). This uracil is subsequently read as thymine by DNA polymerases during DNA replication or repair processes, resulting in a C>T to G>A base‐pair conversion.^[^ ^17^, ^18^ ^]^ Recent advancements in CBE technology have led to the development of more efficient and versatile BEs. This includes natural cytidine deaminase such as Apolipoprotein B mRNA Editing Catalytic Polypeptide‐like (APOBEC1) is replaced with different members of the APOBEC family, other deaminases, or unnatural lab‐evolved adenine deaminase, such as TadA. Compared to commonly used CBEs, TadCBEs offer comparable or greater on‐target activity, reduced size, and significantly reduced Cas‐independent off‐target editing activity in both DNA and RNA.^[^ ^19^, ^20^ ^]^

ABEs: ABEs utilize adenine deaminase enzymes of purine metabolism to convert adenine to inosine in the Cas9 editing window. During DNA replication or repair, inosine is interpreted as guanine, leading to A>G or T>C changes, depending on which DNA strand is targeted.^[^ ^21^ ^]^ Inosine excision is less efficient in eukaryotic cells; thus, there is no need for a UGI‐like domain in ABE. Several variants of ABE are being developed, including a variant of maximum efficiency, ABEmax, optimized for improved expression through codon modification, and nuclear localization in mammalian cells,^[^ ^22^, ^23^ ^]^ PhieABEs, a PAM‐less ABE that combines an evolved, highly active adenosine deaminase TadA8e with Cas9 nickase variants that recognize broader PAM sequences like NGN or NNN,^[^ ^24^ ^]^ and, ABE 9, featuring V82S/Q154R mutations in TadA8e is compatible with multiple CRISPR systems including SpCas9‐NG and achieves similar or improved editing across a broader editing window at various PAM sites compared to TadA8e.^[^ ^25^ ^]^ The compact size of the smaller ABE (sABE) created by removing HNH and REC2 domain of SpCas9, allows for delivery in vivo using an adeno‐associated virus (AAV) vector.^[^ ^26^ ^]^ The decision to choose the version of BE to be employed for a study should be based on criteria such as the type of desired edit, availability of PAM near the desired edit,  the position of the desired edit, possible bystander conversions, and phenotypic effects associated with it, editing efficiency and purity required, off‐target effects, and available delivery mode.

BE enables the precise introduction of specific base changes in genomic DNA or cellular RNA without relying on the cell cycle and DNA donor templates. It can introduce the 4 transition mutations (C>T, T>C, G>A, and A>G) precisely in a wide range of cell types.^[^ ^14^, ^27^ ^]^ BE has been used to correct various hematological disorders. Specifically, ABE8e has shown promise in altering the SCD mutation into a non‐pathogenic variant, HbG‐Makassar, with an efficiency of up to 80% in HSPCs^[^ ^28^ ^]^ and has been shown to correct severe β0/βE‐thalassaemia mutation in patient cells.^[^ ^29^, ^30^ ^]^ A near‐PAMless SpCas9 variant named SpRY‐ABE8e corrected a predominant beta‐thalassemia mutation, IVS1‐110 (G>A) in patient‐derived HSPCs.^[^ ^31^, ^32^ ^]^ Base editing is also being investigated for the correction of blood disorders caused by point mutations, such as Fanconi anemia and inherited platelet disorders.^[^ ^33^, ^34^ ^]^

The BEACON trial (NCT05456880) and BE CAR‐7 (NCT04572308) represent advanced applications of BE in hematological disorders. The BEACON trial utilizes ABEs to edit the γ‐globin promoter for its activation and to alleviating severe SCD.^[^ ^35^ ^]^ BE CAR‐7, on the other hand, employs CBEs to edit TRBC1/2, CD7, and CD52 genes, with the goal of creating universal, off‐the‐shelf chimeric antigen receptor (CAR) T cells for treating T‐cell Acute Lymphoblastic Leukemia (T‐ALL).^[^ ^36^ ^]^ In the realm of non‐hematological applications, the heart‐1/VERVE‐102 (NCT05398029) study stands out as an advanced use of base editing technology. This trial uses ABEs delivered via lipid nanoparticles to edit the PCSK9 gene for the reduction of cholesterol levels and decrease the risk of familial hypercholesterolemia through in vivo gene editing.^[^ ^37^ ^]^

Currently, these techniques are unable to facilitate the 8 transversion mutations (C>G, C>A, G>T, G>C A>C, T>A, T>G, and A>T), including the critical T>A to A>T mutation necessary to correct the primary cause of SCD (HBB‐E6 V),^[^ ^21^, ^38^ ^]^ as well as the A>C mutation in the RB1 gene that leads to retinoblastoma and the G>C mutation associated with Huntington's disease.^[^ ^39^ ^]^

Moreover, there lacks a method that is capable of executing precise deletions, for example, the elimination of the 4 bp duplication responsible for Tay‐Sachs disease,^[^ ^40^ ^]^ or targeted insertions, like 3 bp insertion essential to address the most prevalent cause of cystic fibrosis (F508del).^[^ ^41^ ^]^ Thus, a significant percentage of currently annotated human pathogenic variants cannot be corrected by the BE technology and this need has resulted in the development of the PE system.^[^ ^13^ ^]^

## 3 Prime Editing (PE)‐ A Versatile Genome Editing Tool for all 12 Base Conversions

**Figure 1:**
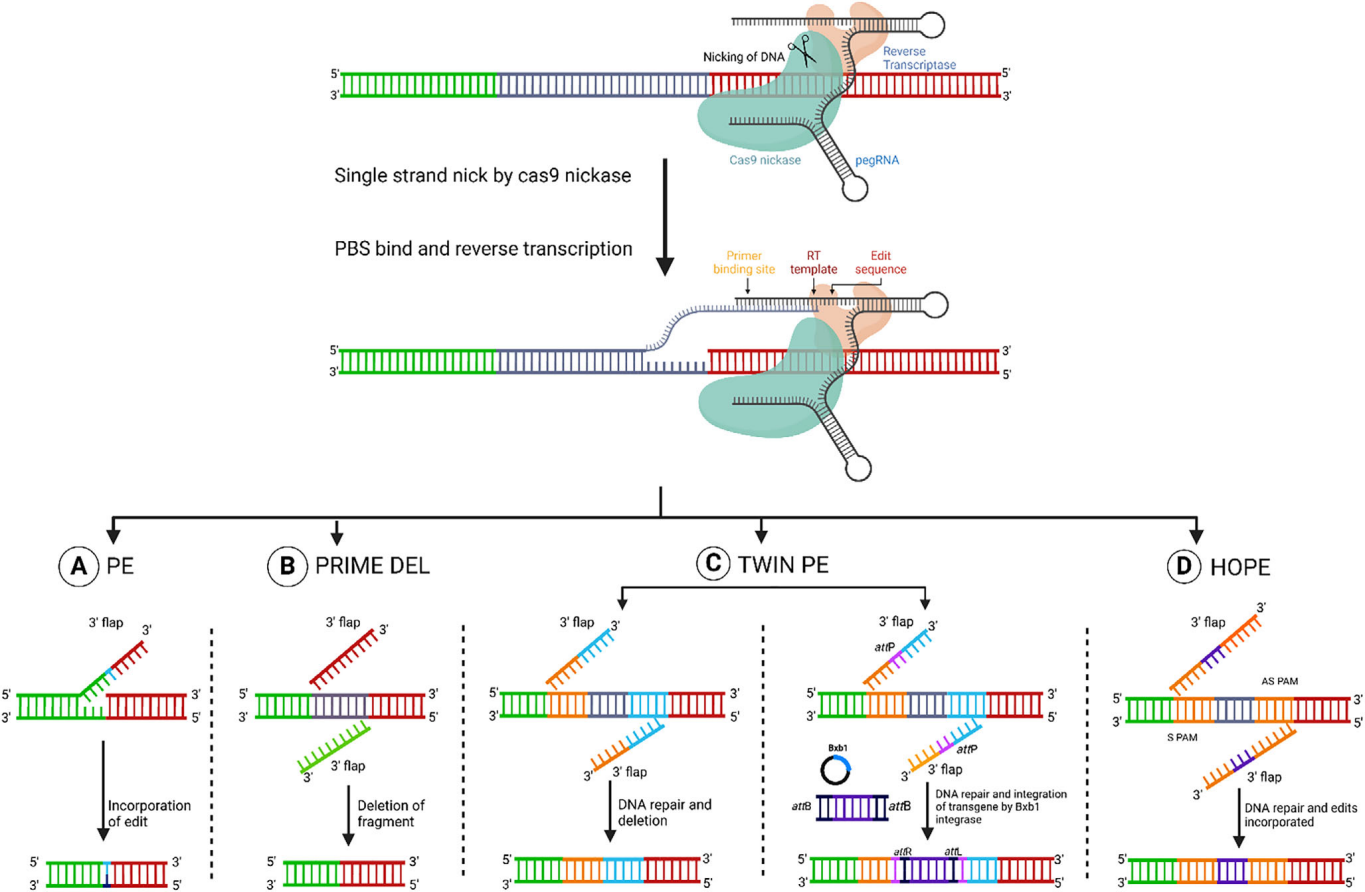
Nickase‐based approach for the deletion and integration of genes. A) Prime editing: This design employs pegRNAs that target DNA strands. The pegRNA specifies the sites to be cut and the 3′ flap that contains the edited sequence integrated into the DNA by the DNA repair mechanism. The regions where DNA is edited are indicated by blue in the 3′ flap regions of homology shown in green and red boxes (red to red and green to green). B) PRIME DEL: This design employs pairs of pegRNAs that target opposite DNA strands. Each pegRNA specifies the sites to be cut at both ends of the desired deletion and includes a 3′ flap that complements the region targeted by the other pegRNA. The regions where DNA is excised are indicated by shaded gray boxes, with the 3′ flap regions of homology shown in green and red (red to red and green to green). C) Twin Editing: This method allows for the flexible deletion of sequences at arbitrarily chosen positions between the 2 nick sites and can also insert a sequence, replacing the endogenous sequence with the Bxb1 attB sequence. The gene of interest with attP flanked sequence at 5′ and 3′ specifically inserted to the target site has an attB site by Bxb1 integrase (plasmid). The excised DNA regions are marked by shaded gray boxes, with the 3′ flap regions of homology indicated in orange and blue. The gene of interest flanked by attB sites is shown in a violet box, while the attP site is indicated by a purple box. D) HOPE: This approach involves base substitutions using paired pegRNAs, with the S‐pegRNA and AS‐pegRNA encoding the same edits in the sense and antisense DNA strands, respectively. The edited regions of DNA are indicated by shaded gray boxes, with the sequence containing the desired edit shown in violet, and the 3′ flap region of homology highlighted in the orange box. Figure created with Bio Render.com.

PE can achieve all 12 possible base conversions, overcoming the limitations of BE, and utilizes an engineered reverse transcriptase (RT) along with Cas9 nickase (Cas9H840A), which selectively nicks the DNA strand complementary to the gRNA target site. The PE guide RNA (pegRNA) is specially engineered with a 3′ end extension that includes a reverse transcription template (RTT) containing the desired edit and a primer binding site (PBS) that can specifically bind to the exposed single‐stranded DNA at the nicked site. The pegRNA directs Cas9 nickase to the target, creating a single‐strand break. The PBS then binds to the target, enabling the RT to synthesize a new strand from the RTT. DNA repair machinery processes the resulting 5′ and 3′ flaps, incorporating the edited sequence into the genome (**Figure** **1**a).^[^ ^13^ ^]^

To improve the efficiency of PE, an engineered RT enzyme derived from Moloney murine leukemia virus (M‐MLV RT) was introduced and named PE‐2. The modifications improve thermostability, processivity, DNA‐RNA substrate affinity, and deactivate RNase‐H activity.^[^ ^42^ ^]^ PE1 editing efficiencies at various loci ranged from 0.7% to 5.5% when transversion point mutations were introduced. PE2 increased the editing efficiency by 1.6‐ to 5.1‐fold.^[^ ^42^ ^]^ Further enhancements led to the development of PE3, which introduced an additional nickase on PE2, achieving higher efficiencies for specific insertions.^[^ ^43^ ^]^ Incorporation of a human codon‐optimized RT, C‐terminal c‐Myc nuclear localization signal (NLS), and mutations enhancing nCas9 activity resulted in another variant called PEmax and it demonstrated a 2.5‐fold enhancement in base substitution compared to PE2.^[^ ^43^ ^]^

To further improve the editing efficiency, additional functional domains were effectively fused to PE. Examples include incorporating a Rad51 DNA‐binding domain, known as hyPE2,^[^ ^44^ ^]^ or, a chromatin‐modulating peptide fused to PE3 to increase chromatin accessibility known as CMP‐PE3‐V1,^[^ ^45^ ^]^ or, the fusion of a dual DNA repair‐related peptide to increase the expression of PE2 known as IN‐PE2.^[^ ^46^ ^]^ Modifying the pegRNA design has also proven effective. spegRNA, which carries silent mutations to enhance base pairing improves the base substitution efficiency of PE3 by 353‐fold.^[^ ^47^ ^]^ Altering the pegRNA secondary structure to create an altered pegRNA (apegRNA), significantly enhanced PE's efficiency in indel editing by up to 10.6‐fold. The combined strategy of spegRNA and apegRNA showed further editing improvements compared with the PE3 system. These innovative PE architectures and guide RNA designs enable small indels and base editing without the need to generate DSBs or utilize donor DNA.^[^ ^47^ ^]^

Subsequent independent studies across diverse model organisms have verified the lower off‐target efficiency of PE. For instance, in mouse embryos, the off‐target frequency was less than 0.1%, and in organoid lines, no observable off‐target mutations were detected.^[^ ^48^, ^49^ ^]^

PE has shown promising results in the treatment of various genetic disorders, particularly hematological conditions. In SCD, PE successfully edited the sickle mutation to wild‐type HBB in the patient HSPCs with an efficiency ranging from 15% to 41%.^[^ ^50^ ^]^ Importantly, these edited HSPCs maintained their editing efficiency for 17 weeks when transplanted into NBSGW mice, with erythroblasts producing sufficient HBB for potential clinical benefits.^[^ ^51^ ^]^ In vivo, transduction of PE expressing helper‐dependant adenovirus in the SCD mouse model (CD46/Townes mice) resulted in 40% correction of sickle mutation.^[^ ^50^ ^]^

Similarly, PE has been demonstrated to be effective in treating chronic granulomatous disease (CGD). In this case, PE corrected the delGT mutation in p47phox‐deficient patient HSPCs, restoring p47phox protein expression and NADPH oxidase activity to ≈80% of normal levels in myeloid cells.^[^ ^52^ ^]^ PE technology has also shown promising results for the modification of CD123 epitopes in HSPCs. This approach can be utilized to distinguish donor‐transplanted cells from recipient cells and to overcome the fratricide effect in targeted therapies for acute myeloid leukemia (AML).^[^ ^53^ ^]^

Beyond hematological disorders, PE has been used in Duchenne muscular dystrophy (DMD) mouse models to restore dystrophin and improve muscle function in vivo.^[^ ^54^ ^]^ Also, the correction of the CFTR gene mutation in the cystic fibrosis patient‐derived lung cells and organoids, led to restoring the CFTR protein's glycosylation, localization, and function.^[^ ^55^ ^]^

Despite its potential, PE has some limitations. Delivering PEs is difficult because of their large size and linear pegRNAs tend to be unstable and prone to nuclease degradation. Additionally, the 5′ spacer of the sgRNA within the pegRNA can bind to the 3′ PBS, causing pegRNA circularization, which disrupts reverse transcription by the RT.^[^ ^56^, ^57^ ^]^

Compared to cell lines, the reduced efficiency of PE in primary cells is primarily due to challenges with cargo delivery, differences in DNA repair mechanisms, and Toll‐like receptor responses to the PE mRNA. The maximum editing efficiency of PE observed is 40% in human hepatocytes with the help of DNA repair modulating small molecules and <50% in T cells with small RNA binding protein.^[^ ^58^, ^59^, ^60^ ^]^ Also, PE can induce unintended large deletions in the genome.^[^ ^61^ ^]^

Attempts have been made to address these challenges. This includes the development of a dual AAV system, that effectively delivers the components of PE separately, chemical modification at the 3′ end of pegRNA, and optimizing the length to stabilize the pegRNA and co‐delivery of factors such as a modified MLH1 variant (MLH1dn) that suppresses mismatch repair to improve the editing efficiency.^[^ ^62^, ^63^, ^64^ ^]^ Perhaps the most exciting of these developments are the new variants of PE that significantly expand the capabilities and efficiency of this innovative genome‐editing technology. Here, we discuss some of the newly developed technologies based on PE (**Table** **1**).

**Table 1:** Recent advances in gene editing technologies.

Editing Tool	Components	Percentage of Edits (Highest)	Target	Cellular Model Tested
PRIME DEL^[^ ^65^ ^]^	Cas9 nickase, RTase and pair of pegRNA (RTT homologous to the complementary strand cut site)	3%	HPRT1, 1 kb deletion	HEK293T
Twin PE^[^ ^66^ ^]^	Cas9 nickase, RTase and pair of pegRNA (RTT complimentary to each other)	16%	PAH, 108 bp insertion	HEK293T
HOPE^[^ ^67^ ^]^	Cas9 nickase, RTase and pair of pegRNA (sense and antisense complimentary to each other)	32.6%	HEK site 3	HEK 293T
PEDAR^[^ ^68^ ^]^	Cas9 nuclease, RTase and pair of pegRNA (RTT complimentary to each other)	18.4%	8 kb deletion in the HEK site 3	HEK 293T and Mice hepatocyte
PETI^[^ ^69^ ^]^	Cas9 nuclease, RTase, and pair of pegRNA (RTT homologous to the complementary strand in targeted chromosomes)	43%	HEK 3 translocation	HEK 293T
sPE^[^ ^70^ ^]^	Cas9 nickase, RTase, LPET RNA/PET RNA, MCP‐MS2 and gRNA	74.2%	VEGFA	HEK293T
DPE^[^ ^56^ ^]^	Cas9 nickase, RTase, DPET RNA, MCP‐MS2 and gRNA	60%	FANCF	HEK293T
CE^[^ ^71^ ^]^	Cas9 nickase, Ecklenow (DDP), clk DNA, HuHe (Tethering domian) and gRNA	26.6%	VEGFA	HEK 293 T Cells
PASTE^[^ ^72^ ^]^	Cas9 nickase, RTase, Bxb1 integrase and atgRNA	15%	ACTB	K562, T cells, and hepatocyte
CREAT^[^ ^73^ ^]^	Cas9 nickase, ORF1p, ORF2p and CREAT mRNA	≈2%	GFP insertion in AAVS1 locus	Huh7 and HEK293T
CPE^[^ ^74^ ^]^	Cas12a nuclease, RTase, Circular RNA, MCP‐MS2	40.75%	HEK site 2	HEK 293T cells
Rep editing^[^ ^75^ ^]^	saCas9 nuclease, Rep RNA, Rad52, and Pol θ	40%–80%	AAVS1	HEK 293 cells

### 3.1 PRIME‐DEL for Precise Excision of Large DNA Fragments

PRIME‐DEL uses a pair of pegRNAs that target opposite DNA strands, allowing it to specifically nick both the sites and the repair outcomes, providing greater control over the editing process compared to the conventional Cas9 paired‐gRNA approach.^[^ ^65^ ^]^ PRIME‐DEL achieved significantly higher precision compared to conventional Cas9 and gRNA pairs, particularly for deletions of up to 10 kb (Figure 1b), with 97% of cells lacking indel errors at post‐deletion junctions. In contrast, the Cas9/paired‐gRNA strategy exhibited only 42% of the reads lacking indel errors for the same deletions.^[^ ^65^ ^]^ Although PRIME‐DEL may exhibit lower efficiency and can still generate minor indels at the target sites, it offers a means for accurate and adaptable programmed genomic deletions, including in‐frame deletions and applications in epitope tagging.^[^ ^65^ ^]^ Large genomic deletions in the beta‐globin locus using paired sgRNA and Cas9 have been shown to mimic the hereditary persistence of fetal hemoglobin (HPFH) mutations but with indel errors at both the cut‐sites.^[^ ^76^, ^77^ ^]^ PRIME‐DEL has the potential to improve the precision of such genomic deletion strategies for β‐hemoglobinopathies gene therapy.

### 3.2 Twin Prime Editing (Twin PE) for Precise Insertion of Large DNA Fragments

The TwinPE strategy uses 2 pegRNAs that generate 2 3′ flaps containing the desired edits that are complimentary to each other. As a result, strand invasion of the target site is not required, nor is it necessary for the edit to be copied to the complementary DNA strand (Figure 1c). It eliminates the requirement of homologous DNA sequence in the pegRNA templates, allowing flexibility in choosing template sequences and enabling larger insertions using shorter RTTs.^[^ ^66^ ^]^ TwinPE replaced a 90 bp endogenous sequence with a Bxb1 integrase attB site and an attP attachment sequence, deleted a 780 bp in exon 51 of the DMD gene, inverted a 40 kb sequence between the IDS and IDS2 sequences and inserted a 5.6 kb transgene at various genomic sites.^[^ ^66^ ^]^ However, the precise design principles governing the performance of TwinPE pegRNAs are yet to be elucidated. Hematological diseases such as Wiskott‐Aldrich syndrome^[^ ^78^ ^]^ are associated with at least 300 mutations in their loci and techniques such as Twin PE can be applied to replace defective genes.^[^ ^66^ ^]^

### 3.3 Homologous 3′ Extension Mediated Prime Editor (HOPE) for Enhanced Editing Purity

HOPE uses a sense pegRNA (S‐pegRNA) and antisense pegRNA (AS‐pegRNA) to edit both strands of a target DNA locus simultaneously. The pegRNA pairs are configured with inward‐orientated PAMs (PAM‐in configurations) to prevent potential editing conflicts between the 2 strands (Figure 1d).^[^ ^67^ ^]^ It is crucial to avoid overlapping protospacer sequences, as edits on one allele could create mismatches that hinder the editing of the other allele.

In HEK293T cells, HOPE increased the efficiency of the G >T transversion edit in the PRNP gene compared with PE3. HOPE's editing capacity was on par with the optimal PE3 for C>G editing in PDCD1 and A>C editing in METTL3.^[^ ^67^ ^]^ Additionally, HOPE achieved the desired insertion ratios averaging 32.6% insertions, and showed over 30% efficiency in 8 bp deletions. At the human FANCF and EMX1 loci, HOPE reduced undesired indel formation by 11‐ and 6‐fold, respectively, compared to the optimal PE3, without significantly increasing off‐target effects. These findings suggest that HOPE enhances the editing efficiency minimizing the risk of undesired indel formation and off‐target effects.^[^ ^67^ ^]^

### 3.4 PE‐Cas9‐Based Deletion and Repair (PEDAR) for Deletion and Replacement of large DNA Fragments

**Figure 2:**
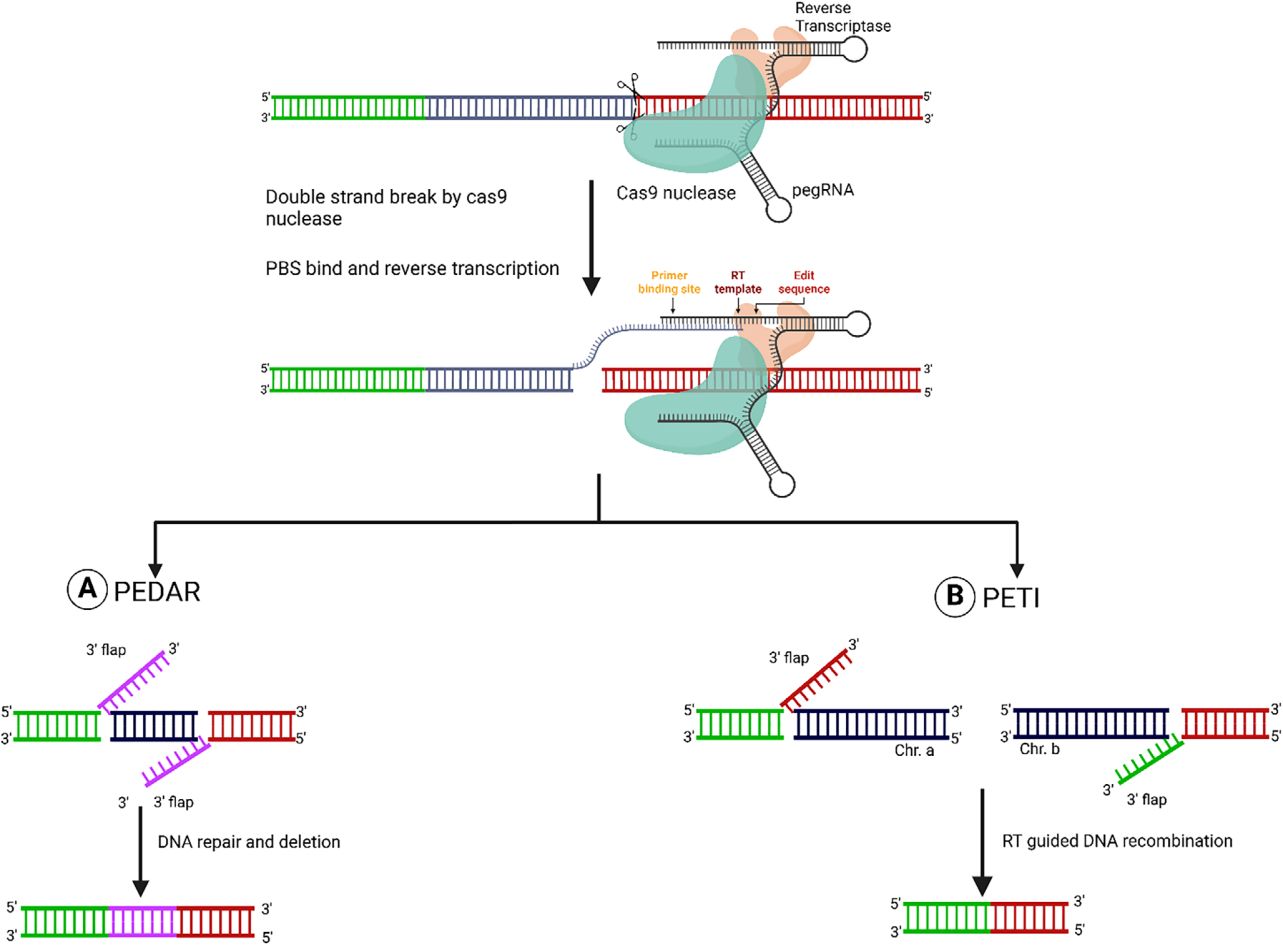
Nuclease‐based approach for the deletion and translocation of genes. A) PEDAR: The Cas9 nuclease generates a double‐strand break followed by 2 3′ flaps created by paired pegRNAs that target the complementary DNA strands (pegF and pegR). The 2 3′ flaps generated by these pegRNAs are complementary. The sequence deleted in between the flap with excised by the DNA repair mechanism. The regions where DNA is excised are indicated by dark blue boxes, with the complementary 3′ flaps shown in the purple box. B) PETI: Similar to PEDAR, the Cas9 nuclease induces a double‐strand break followed by 2 3′ flaps created by paired pegRNAs that target different chromosomes and are complementary to the sequence of other target sites. This results in the translocation of chromosomes, indicated by the green and red 3′ flaps showing the regions of homology (red to red and green to green) to the targeted site. Figure created with Bio Render.com.

Traditionally, PE utilizes Cas9 nickase. In the PEDAR approach, an active Cas9 nuclease is fused with RT instead of Cas9 nickase. This approach, similar to Prime Dl, targets both DNA strands simultaneously with 2 pegRNAs (pegF and pegR), creating 2 DSBs that remove the DNA segment between the cuts. (**Figure** **2**a).^[^ ^68^ ^]^

PEDAR achieved an efficiency of 2.6% for a 991 bp deletion and integration of 18 bp at the HEK3 genomic locus in HEK293T cells. For large deletions ranging from 8 to 10 kb, PEDAR demonstrated efficiencies of up to 18.4%.^[^ ^68^ ^]^ When PEDAR was hydrodynamically injected into the liver, to delete ≈1.3 kb and insert a 19 bp sequence into exon 5 of the FAH gene, which causes FAH deficiency (tyrosinemia‐I),^[^ ^79^ ^]^ a correction rate of 0.76% ± 0.25% is observed in vivo.^[^ ^68^ ^]^ In comparison to PRIME‐Del, PEDAR appears to exhibit higher error rates, introducing increased proportions of direct deletion and imperfect deletion and insertion events.^[^ ^65^, ^68^ ^]^ However, PEDAR has the potential to program targeted deletion and insertion in quiescent hepatocytes within the mouse liver, particularly in scenarios where HDR is not conducive.^[^ ^80^ ^]^

### 3.5 Prime Editor Nuclease‐Mediated Translocation and Inversion (PETI) for Specific Chromosome Translocation

Precise chromosomal rearrangements, including translocations and inversions, have traditionally been challenging. Using paired pegRNAs in conjunction with PE2 nuclease (Cas9 nuclease‐RT fusion), PETI facilitated targeted chromosomal rearrangement (Figure 2b). PETI functions by generating DSBs at 2 separate sites.^[^ ^69^ ^]^ These breaks are then repaired by rejoining the DNA ends in a new configuration, with the process guided by 3′ overhangs produced through reverse transcription, which is complementary to the other target sites. Compared to the widely used CRISPR‐Cas9 system, PETI demonstrates superior accuracy in generating reciprocal translocations between HEK3 and HEK4 genomic sites.^[^ ^69^ ^]^

Beyond targeted translocations, PETI has shown promise in inducing cancer‐associated inversions, such as in the EML4‐ALK V2 fusion, and also allows for precise editing of cancer‐associated translocations by insertion or removal of specific sequences at the junctions of rearranged genomic breakpoints. This includes the ability to insert small sequences or even a 40 bp attP sequence at the breakpoint junction, which can be useful for site‐specific recombination.^[^ ^81^ ^]^ Despite the lower translocation efficiency in some cases, such as cancer‐associated NPM1‐ALK translocation, the programmable nature of PETI opens a wide range of potential applications. These include gene tagging and the insertion of epitope sequences, which could prove invaluable in various fields, such as disease modeling and correction.^[^ ^69^ ^]^

### 3.6 Split Prime Editing (sPE) for Stability and Efficiency

**Figure 3:**
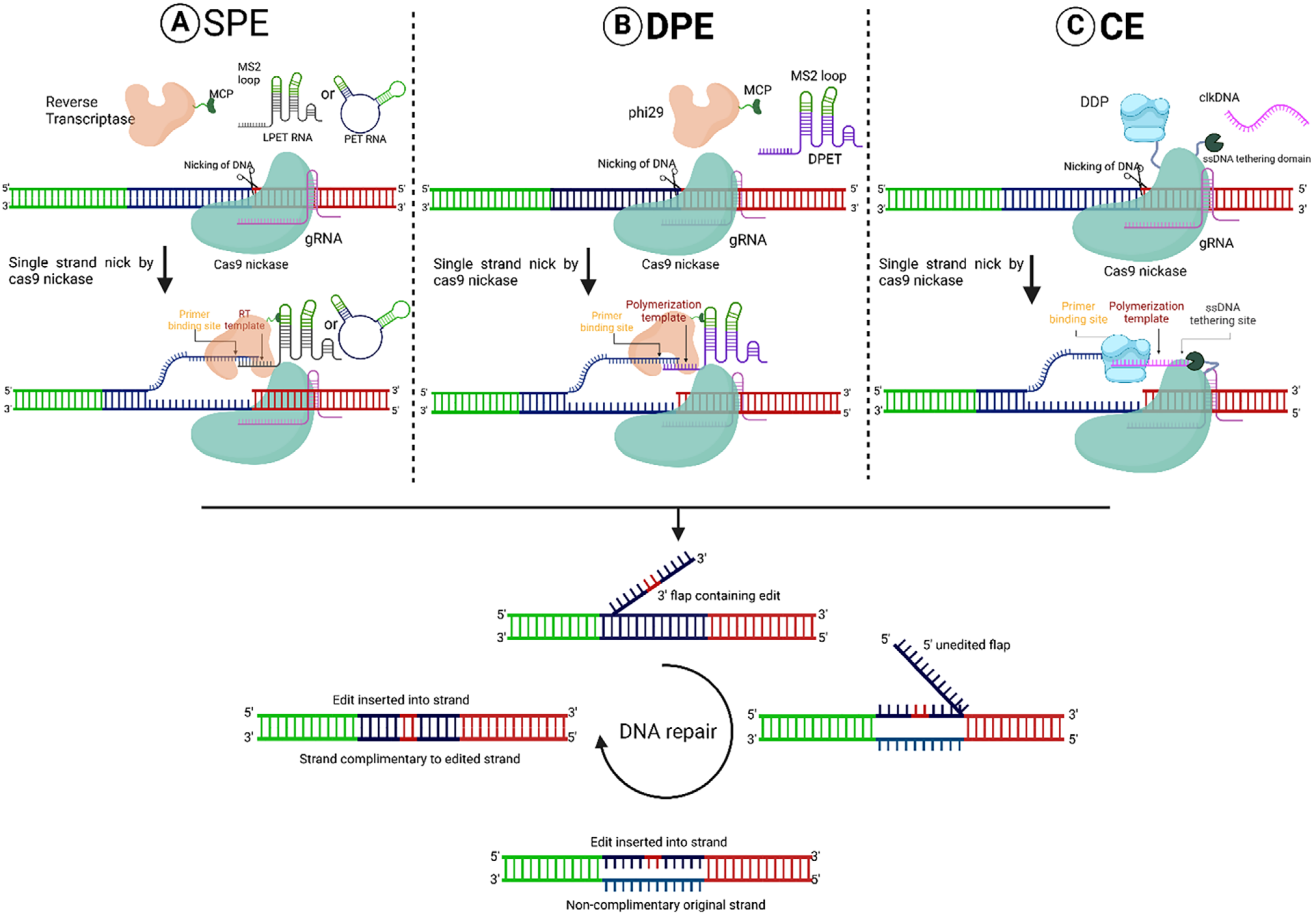
DNA polymerase‐assisted gene editing. A) SPE: This prime editing approach involves splitting the reverse transcriptase from Cas9. The reverse transcriptase (RT) and template RNA (LPET and PET) are tethered by MCP and the MS2 loop. LPET/PET anneals to the nicked DNA strand and serves as the template for RT. A single‐strand nick in the DNA by Cas9 nickase drives reverse transcription by RT, thereby incorporating the desired edit into the target site. B) DPE: In this design, RT is replaced by MCP‐tethered phi29, which binds to the DPET via MS2. The DPET anneals to the nicked DNA strand and serves as the template for phi29 polymerase (a DNA polymerase). DPET encodes the primer‐binding site and the prime‐editing template. C) CE: This system consists of a Cas9 nickase, a DNA‐dependent DNA polymerase, and a single‐stranded DNA (ssDNA) tethering domain (HUHe) paired with a guide RNA (gRNA). The clkDNA is a single‐stranded DNA oligonucleotide that encodes a PBS, a PT, and an HUHe recognition site, serving as a template for the DNA polymerase. Polymerization of the template strand and subsequent DNA repair mechanisms lead to the incorporation of the desired edit into the target strand. Figure created with Bio Render.com.

The large size and complex domain arrangements of PE present challenges in delivering them into cells, limiting editing efficiency. The sPE approach was designed to address these limitations by separating the 2 key components of the PE machinery, nCas9‐RT, and pegRNA, into 4 components, namely nCas9, RT, circular RNA RTT, and sgRNA, to enhance stability and efficiency (**Figure** **3**a). A notable feature of sPE is the use of a circular prime editing template RNA (petRNA) to act as a PBS/RTT component. This circular structure provides increased stability against exonuclease degradation and prevents intramolecular spacer/PBS pairing.^[^ ^70^ ^]^ sPE demonstrates editing efficiency comparable or superior to that of traditional PE systems. This includes editing at various endogenous such as VEGFA, FANCF, and HBB, and in vivo editing for tumor induction and correcting tyrosinemia‐causing mutation.^[^ ^70^ ^]^ The modularity and compact size of sPE allow mRNA and RNP versions of sPE and easy packaging into lipid nanoparticles. The AAV, mRNA, and RNP approach for delivery enhances cellular delivery.^[^ ^70^ ^]^ Further investigations are required to ascertain whether the RT (whether tethered or untethered) can use RNA‐RNA or RNA‐DNA hybrids, potentially leading to undesired genomic integration events^[^ ^82^ ^]^ and to investigate the potential off‐target effects of sPE.^[^ ^70^ ^]^

### 3.7 DNA Polymerase Editing (DPE) for Better Efficiency

RT‐based PE efficiently can lead to precise edits in the genome, but complex cellular events introduce challenges. The abundance of cellular RNAs can lead to unintended byproducts, including self‐primed cDNA; this unintended cDNA can interact with genomic DNA, potentially causing recombination events or interfering with the editing, although evidence of such genome‐altering phenomena during prime editing is lacking. Despite the presence of NLS in the PE, any residual or transiently cytoplasmic RT could generate cytoplasmic cDNA, potentially triggering innate immune responses via the cGAS–STING pathway.^[^ ^83^ ^]^

DPE uses DNA‐dependent DNA polymerase (phi29) instead of RT enzymes and also uses a linear prime editing template (LPET) or DNA‐containing template (DPET) instead of engineered pegRNA to improve the stability of the template (Figure 3b).^[^ ^56^, ^84^ ^]^ DPE has demonstrated an editing efficiency of up to 60% in various cell lines.^[^ ^56^ ^]^ The versatility of prime editing polymerase modules and template chemistries has the potential to facilitate various applications and potentially improve the specificity of the approach.^[^ ^56^ ^]^

### 3.8 Click Editors (CE) for stability, Precision, and Cost‐Effectiveness

Click editors also use DNA‐dependent DNA polymerases; a Klenow fragment from *E. coli* DNA polymerase I (EcKlenow) fused with nCas9. In addition, they also contain an ssDNA recruitment domain, HUH endonucleases (HUHes) tethered to nickase. The second component in this system is a click DNA (clk DNA) which is a single‐stranded DNA, containing recognition sequences for binding with HUHe, a polymerization template (PT) housing a specific edit, and PBS exhibiting homology to the nicked non‐target strand (NTS) of the designated target site (Figure 3c). The “click”‐like bioconjugation between HUHe and clk DNA assembles the editing machinery to mediate the editing, and the third component, sgRNA, targets the machinery to specific sites in the genome.^[^ ^71^ ^]^

Clk editing demonstrates precise polymerization, displays strong substrate processivity, and is likely to be enzymatically active in almost any cell type owing to its high affinity for dNTP. Also, it is amenable to user‐specified DNA oligonucleotide templates, presenting advantages in simplicity, scalability, cost‐effectiveness, and additional silent mutations.^[^ ^71^, ^85^ ^]^ The efficiency of precise editing with CE was comparable to that of PEs. In addition, there was no detectable incorporation of the gRNA scaffold or heteroduplex unwinding HUHe site. This observation suggests the existence of 2 potential mechanisms to mitigate undesired insertions: first, the inherent separation of the template from the gRNA in CEs, and second, the covalent attachment of the heteroduplex unwinding HUHe to the clk DNA. These findings provide valuable insights into the mechanisms that effectively limit unwanted insertions in CE compared with PE.^[^ ^71^ ^]^

### 3.9 Programmable Addition via Site‐Specific Targeting Elements (PASTE) for Efficient Integration

**Figure 4:**
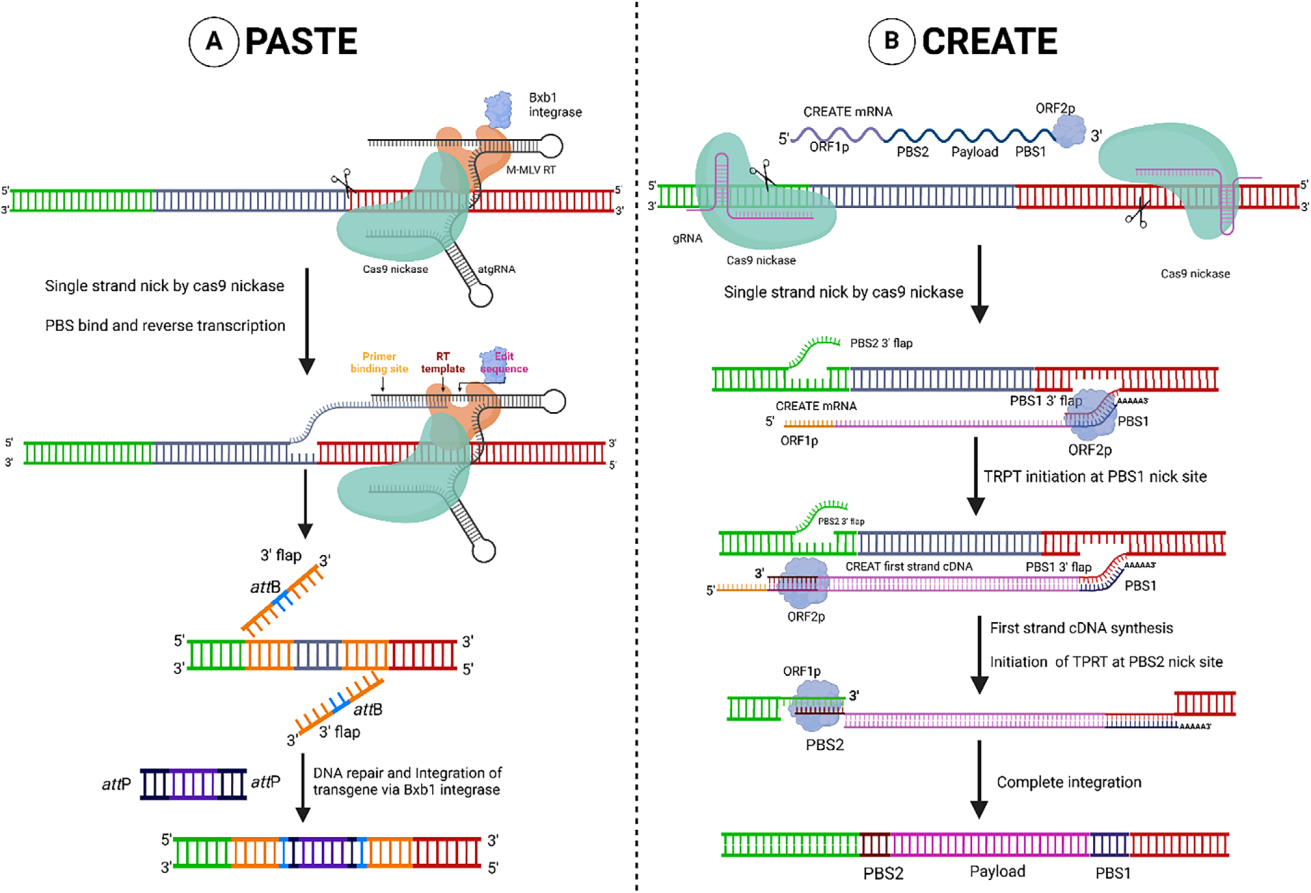
RT and transposable element‐based techniques for efficient integration. A) PASTE: Prime editing allows for the insertion of the attB sites in the target sequence by atgRNA (gRNA+ RT with attB site), and the gene of interest flanked by the attP sequence is inserted into the target site by the Bxb1 integrase. In the diagram, the gene of interest is shown in the violet box, the attP site in the dark blue box, and the attB site in the light blue box. B) CREATE: The CREATE system produces ORF1p and ORF2p from CREATE mRNA, which are co‐assembled into a ribonuclear complex with the CREATE mRNA. Cas9H840A and sgRNAs induce non‐target strand nicking. The ORF1p RNA‐binding protein aids in the formation of the RNP complex. The DNA 3′‐flap hybridizes with PBS1 to prime the first strand of cDNA synthesis mediated by the ORF2p RT domain, while the second strand synthesis is primed by PBS2, which hybridizes with the reverse‐transcribed PBS2. In the diagram, the PBS1 binding site is shown in the blue box, and the PBS2 binding site in the brown box. Figure created with Bio Render.com.

Natural transposable element systems offer efficient genome integration but lack programmability.^[^ ^86^ ^]^ These elements, including various integrase and transposase families, insert multiple copies of donor sequences at semi‐random sites, often TA dinucleotides, across the genome.^[^ ^87^ ^]^ PASTE is an innovative gene integration approach that combines the precision of CRISPR‐Cas9 with the efficiency of site‐specific recombination. This method employs CRISPR‐Cas9 nickase fused to an RT and serine integrase to direct the insertion of attP‐flanked sequences into a target site containing a corresponding attB attachment site (**Figure** **4**a).^[^ ^87^ ^]^

The attB site is introduced into the genome using an attachment site‐containing guide RNA (atgRNA) with a truncated attB sequence.^[^ ^72^ ^]^ PASTEv3‐ an improved version of PASTE developed by engineering integrase, BxbINT, optimizing guides, linkers, and atgRNA, accurately integrated templates up to 36 kb with efficiencies ranging from 10% to 20% in ACTB and LMNB1 loci. PASTE has demonstrated versatility by facilitating insertions at multiple endogenous sites with various cargo sizes, potentially accommodating over 99.7% of human cDNAs. In human primary hepatocytes, PASTE achieved ≈5% gene integration at the ACTB locus. PASTE shows editing efficiencies comparable to or better than those of the HDR‐based integration.^[^ ^72^ ^]^ The potential off‐target effects of the BxbINT integration into pseudo‐attB sites within the human genome require further investigation and mitigation strategies.^[^ ^72^ ^]^

### 3.10 The CRISPR‐Enabled Autonomous Transposable Element (CREATE) for Efficient Integration

CREATE, leverages a CRISPR system that incorporates a human transposable element known as LINE1 (L1). L1 can efficiently reverse‐transcribe large mRNA sequences into cDNA and integrate them into the human genome. The replication of L1 involves a bicistronic mRNA that encodes 2 proteins: RNA‐binding protein (ORF1p) that interacts with L1 mRNA transcripts and ORF2p, which contains an endonuclease (EN) domain that identifies and cleaves the 5′TTTT/AA3' consensus DNA sequence, which pairs with the poly‐A tail of L1 mRNA. The rest of ORF2p forms a groove that tightly binds the RNA: DNA heteroduplex, allowing the RT domain of ORF2p to start the target‐primed reverse transcription (TPRT) reaction for synthesizing the first cDNA strand. It is proposed that the synthesis of the second‐strand cDNA involves an upstream nick and strand exchange via microhomology to initiate TPRT. Additionally, the ORF2p RT domain exhibits high processivity, enabling the reverse transcription of extended RNA sequences.^[^ ^73^, ^88^ ^]^

In the CREATE system, Cas9 nickase is utilized to introduce a single‐strand nick guided by sgRNA. The released DNA 3′‐flap along with PBS1, serves as the template for ORF2p to begin synthesizing the first cDNA strand. Following this, PBS2 pairs with the 3′ flap, released by sgRNA2 nicking, triggers L1 synthesis and integration of the second strand (Figure 4b).^[^ ^73^ ^]^ Using CREATE a 1.1 kb GFP cassette was successfully integrated into multiple mammalian cell lines with an efficiency of ≈2%. To further enhance the CREATE gene delivery system, efforts can focus on optimizing various aspects such as delivery methods and modulation of RNA processing pathways.^[^ ^73^ ^]^ Additionally, since the programmable targeting component and the reverse transcription and integration component (ORF2p) do not need to be covalently connected, this opens the possibility of exploring alternative programmable RNA‐guided DNA endonucleases like TnpB, Fanzor, and Cas12.^[^ ^89^, ^90^, ^91^ ^]^ This exploration could broaden the capabilities of the CREATE system.^[^ ^73^ ^]^

### 3.11 Circular RNA‐Mediated Prime Editor (CPE) for Multiplexing

**Figure 5:**
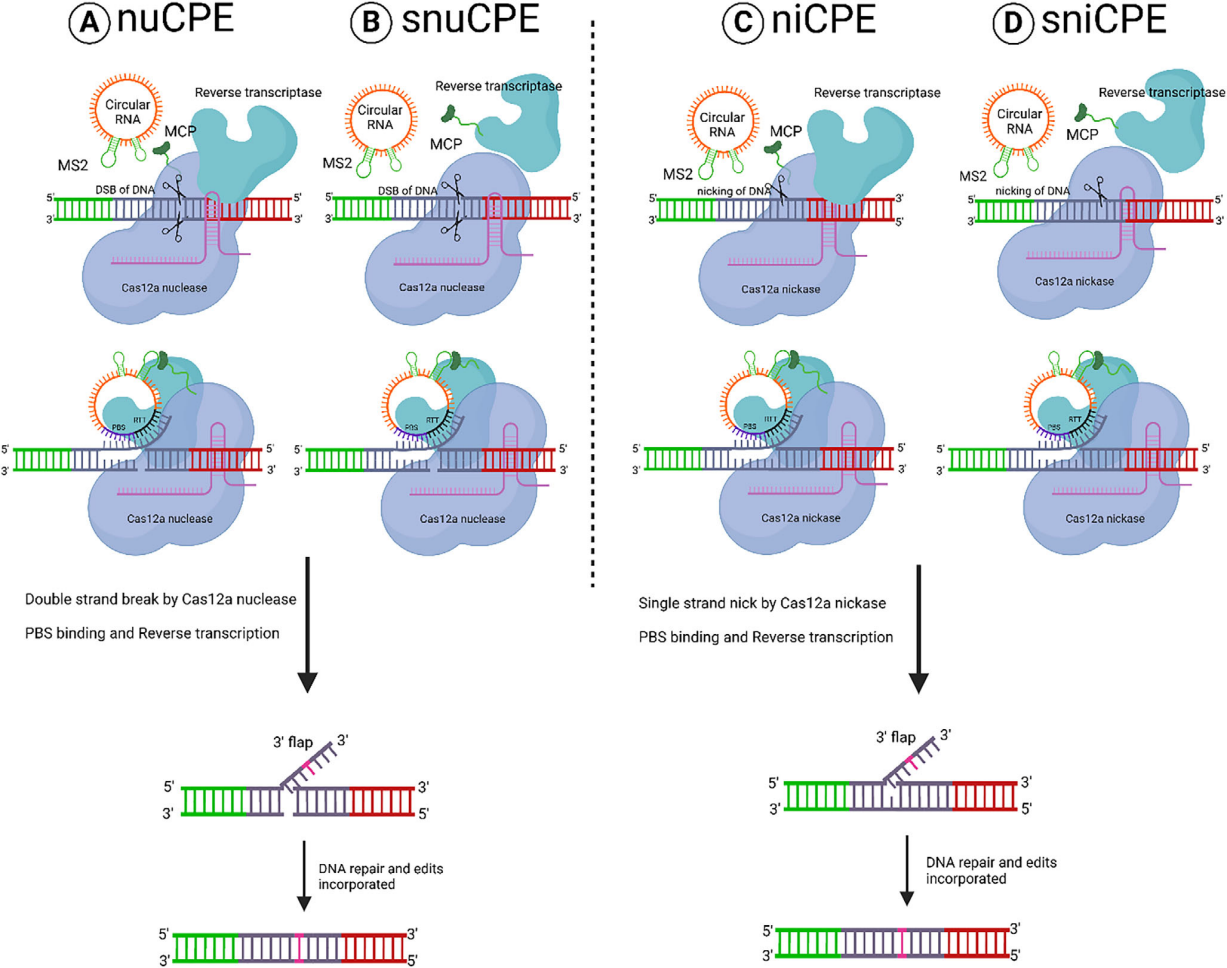
Circular RNA‐mediated gene editing for stability. The CPE (circular prime editing): A and C) Tethered RT and Cas9: In this system, RT and Cas9 are tethered together. The circular RNA encodes the primer‐binding site (PB, shown in blue box) and the reverse transcription template (RT, shown in black box) tethered to Cas9 via MCP‐MS2 interaction. B and D) Untethered RT and Cas9: here RT and Cas9 are not tethered together. The circular RNA encodes the PB (blue box) and RT (black box), with the RT interacting via MCP‐MS2. (A and B) Cas9 nuclease generates a double‐strand break at the target site, with the reverse transcriptase performing reverse transcription to incorporate the desired edits (pink box). (C and D) Cas9 nickase generates a single‐strand nick at the target site, with reverse transcription performed by the reverse transcriptase to incorporate the desired edits (pink box). Figure created with Bio Render.com.

CPE has 3 components: AsCas12a nickase instead of Cas9 nickase, circular RNA that contains the RTT and PBS components, and sgRNA for targeting. Through MCP‐MS2 interactions, circular RNA was brought near the editing site.^[^ ^92^ ^]^ AsCas12a nickase provides advantages such as reduced size of protein and crRNA, and operation in thymine‐rich genomic regions, circular RNA lacking dissociative ends increased stability compared to linear RNAs. A suite of 4 different CPEs, namely nickase‐dependent CPE (niCPE), nuclease‐dependent CPE (nuCPE), split nickase‐dependent CPE (sniCPE) and split nuclease‐dependent CPE (snuCPE) were developed based on CPE. They employ either a wildtype Cas12 (nuclease‐dependent) or Cas12 nickase (nickase‐dependent) or split versions where RT is expressed separately (**Figure** **5**).^[^ ^92^ ^]^

The CPE showed superior editing performance compared to other PEs, with editing efficiencies reaching up to 40.75% in the tested targets. The One‐CPEs represent an enhancement to the existing CPE system, where 4 different RNAs are expressed under a single U6 promoter. The CPE system may pave the way for achieving high‐efficiency, multiplex editing within a cell.^[^ ^92^ ^]^

### 3.12 RNA‐Templated Repair (Rep) Editing for Efficient Deletion and Insertion

**Figure 6:**
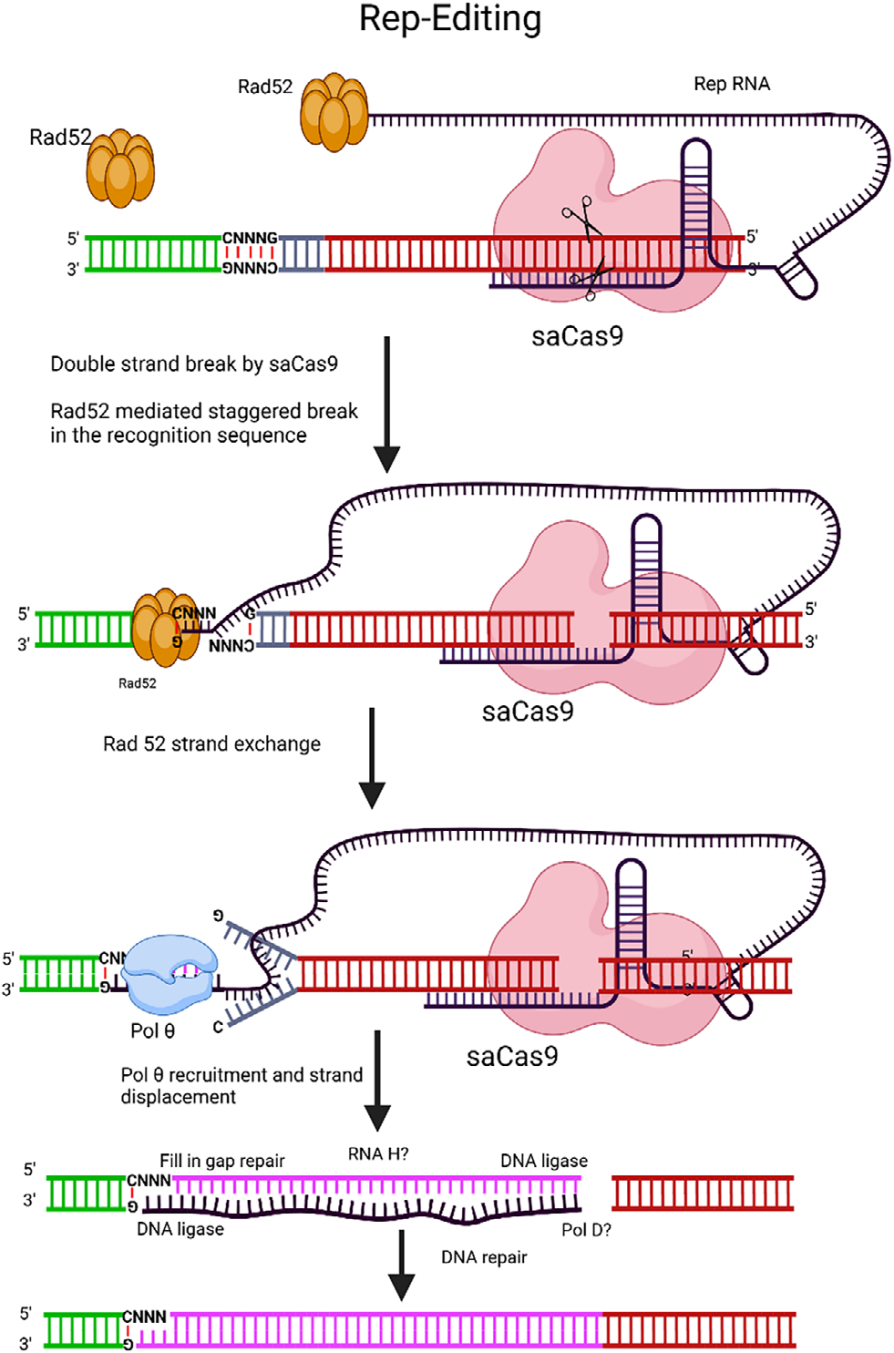
Targeted RNA‐templated mediated gene Editing. Cleavage by TevSaCas9/rep‐gRNA facilitates the recruitment of Rad52, which can bind to RNA or DNA and mediate RNA strand exchange at the Tev cleavage site. This interaction leads to a hybrid RNA structure with a 3′‐OH end, which recruits DNA polymerase θ (Polθ). The repair intermediate is then resolved through a fill‐in‐gap repair process involving DNA ligase and an unidentified DNA polymerase, resulting in the completion of the repair. In the diagram, Rep RNA is shown in the black box, the gene of interest in the pink box, and the DNA flap in the grey box. Figure created with Bio Render.com.

The Rep editing system employs a fusion protein called TevCas9, which combines the GIY‐YIG nuclease domain from the homing endonuclease I‐TevI with Cas9.^[^ ^93^ ^]^ This fusion protein creates sequential nicks on both DNA strands at specific motifs (5′‐CN↑NN↓G‐3′) upstream of the 5′ end of the Cas9 gRNA ‐binding site. A modified rep‐gRNA, which includes an RNA repair sequence fused to the 3′ end of a CRISPR gRNA facilitated the repair (**Figure** **6**).^[^ ^94^ ^]^ The repair is carried out through the activities of Rad52 and DNA polymerase θ (Pol θ) by pairing homologous RNA and DNA through inverse strand exchange activity^[^ ^95^, ^96^ ^]^ and end joining respectively. The repairs are accomplished by a fill‐in‐gap process involving DNA ligase and DNA polymerase.^[^ ^75^, ^97^ ^]^

Rep editing has demonstrated significant editing rates for various therapeutic gene targets, including CFTR mutations associated with cystic fibrosis. Off‐target cleavage analysis of 38 computationally predicted sites with 4 or fewer mismatches to the gRNA and CNNNG motifs revealed no significant off‐target editing.^[^ ^75^ ^]^ This strategy allows for editing of any position between the Tev and SaCas9 cleavage sites up to the rep‐gRNA scaffold region, enabling high efficiency and fidelity of nucleotide changes, deletions, and insertions. The compact sizes of TevSaCas9 and rep‐gRNA fit well within a single AAV viral delivery vector, providing benefits over editing systems that are constrained by their size or require multiple components. Evaluating the rep‐editing specificity and activity in primary and non‐dividing cells could expand their applications.^[^ ^75^ ^]^

## 4 Challenges in Cargo Delivery

Cas9 can be produced as a protein, allowing RNP complex formation before delivery into cells. However, BEs, PEs, and their variants are much larger in size, which challenges recombinant protein production and delivery as RNP cargo. While plasmid‐based approaches are used for cell line studies, mRNA is the preferred cargo for primary cells. Electroporation can deliver both protein and mRNA efficiently and is the most employed delivery system for therapeutically important cells, such as HSPCs. Novel Lipid nanoparticle formulations delivering Cas9 proteins and mRNA for the correction of hematological disorders have been reported to reduce the number of gene editing molecules in cells improving cell viability and safety.^[^ ^98^ ^]^ LNPs are being developed to deliver BE or PE or its variants. mRNA with enhanced LNPs containing the cholesterol analog β‐sitosterol shown to deliver PE mRNA.^[^ ^99^, ^100^ ^]^

Viral vectors, such as AAV, have been shown to deliver both BE and PE, but they present limitations such as continuous expression. HDAd5/35++ vectors have been shown to deliver BE and PEs to HSPCs, and it was recently demonstrated that the prime editing efficiency is up to 40% in vivo in SCD mouse models following the selection of edited cells.^[^ ^50^, ^101^ ^]^ Viral‐like particles (VLP) delivering Cas9 or BE or PE protein are the new addition to the delivery approaches and PE encapsulated VLP (v3 PE‐eVLP) has shown in vivo correction of retinal degeneration in *rd6* mouse model with 15% efficiency and their application in the hematological disorders are expected to increase as it can efficiently transduce hematopoietic cells.^[^ ^102^, ^103^ ^]^

## 5 Safety and Limitations of Current Methods

The therapeutic application of gene editors, particularly for HSPCs, in treating blood disorders necessitates a stringent safety profile. This is crucial because clonal expansion of cells with harmful mutations can significantly impact the entire hematopoietic system, potentially leading to imbalances in cell lineages or the development of malignancies.

DSB‐inducing nuclease‐based gene editors have limitations which include, unintended changes in the expression of off‐target genes, chromosomal translocations between on‐target and off‐target DNA breaks, extensive genome rearrangement (chromothripsis) at the on‐target site, and delayed cell proliferation due to p53 activation.^[^ ^104^, ^105^ ^]^ While nickase‐based gene editors have reduced many of these issues, there remains the risk of single‐strand breaks converting to DSBs during cell replication, potentially affecting genome stability.^[^ ^106^, ^107^ ^]^ Such risk is increased with multiplex editing.^[^ ^108^ ^]^

Nickase‐based gene editors also present new challenges, such as BE showing unwarranted bystander modifications, gRNA‐independent, genome‐wide mutagenesis, and RNA modifications. Additionally, adverse cellular responses may be triggered by reagents and nucleic acid‐processing intermediates involved in BE and PE.^[^ ^108^, ^109^, ^110^ ^]^ The insertion of plasmid backbone sequences at on‐target sites edited by PE3 has been observed.^[^ ^111^ ^]^ Moreover, studies have demonstrated that the reverse transcription of 3′‐extended pegRNAs, specifically the RTT‐PBS sequence, can extend into the tracr‐RNA scaffold. This phenomenon leads to the insertion of scaffold sequences at the PE site, with varying insertion lengths.^[^ ^112^ ^]^

Gene editing proteins and RNAs can trigger innate immune responses. gRNAs with specific structures can activate pathways that lead to the secretion of pro‐inflammatory cytokines and interferons. The introduction of foreign proteins such as Cas9 can elicit adaptive immune responses, including the production of antibodies and the activation of T cells. Such responses can limit the effectiveness of in vivo gene editing by promoting the clearance of the edited cells.^[^ ^113^, ^114^, ^115^ ^]^

Several strategies have been developed to minimize the off‐target effects of genome editing, this includes delivering the editors as RNP that has a short‐expression period in comparison with mRNA, and the use of high‐fidelity variants of Cas, deaminases, and RTs. mRNA engineering has also been shown to reduce the off‐target efforts associated with BE and PE. sgRNA can also be optimized to improve editing precision. This includes chemical alterations to the sgRNA structure, adjusting its length, and increasing its GC content to enhance stability and target binding.^[^ ^116^, ^117^, ^118^ ^]^ Delivery modalities such as lipid nanoparticles can reduce the number of gene editor molecules required for modification, thus reducing off‐target effects due to excess reagents.^[^ ^119^ ^]^ These strategies collectively aim to improve editing precision and minimize unintended effects, underlining the importance of thorough screening and validation of edited cells for therapeutic applications.

The dynamic landscape of gene editing and cell therapy poses distinct regulatory hurdles that require global alignment. As these technologies progress, it is crucial to establish and standardize several key metrics to guarantee their safety, effectiveness, and uniformity across different regulatory environments. The therapeutic efficacy of gene editing can vary significantly depending on the specific disease target gene, making it challenging to define universal benchmarks for editing efficiency. Moreover, the definition of a therapeutic drug product may vary depending on the in vivo or ex vivo gene editing. For in vivo studies, the editing components, together with their delivery vehicles, are classified as therapeutic products. In contrast, for ex vivo, edited cells such as gene‐edited HSPCs for transplantation are considered therapeutic products. Defining appropriate safety studies, off‐target assessment protocols, tracking durations for edited cells, and optimal dosage of editing reagents and edited cells are critical in this regard.

## 6 Conclusion

Enhanced genome editing tools have significantly advanced the field, offering more precise and versatile editing capabilities than traditional CRISPR‐Cas9 approaches. These advancements have paved the way for all possible base‐to‐base conversions as well as insertions, deletions, and translocations, positioning the field to edit virtually any therapeutic target. The recent U.S. FDA approval of the first clinical trial using PE for CGD correction underscores the rapid progress in this area. Several key developments will accelerate the transition from target identification to clinical studies.

These include protein engineering to reduce the size of RT and Cas and enhance catalytic domain activity, mRNA engineering to reduce Toll‐like receptor activation, sgRNA modifications to improve accuracy and stability, new delivery vehicles, including enveloped delivery vehicles, VLPs, and novel lipid nanoparticles capable of delivering RNPs without cellular toxicity. These advancements could potentially reduce the time between diagnosis and genetic treatment for rare diseases that require rapid intervention.

Single editors performing multiple functions are expected to emerge soon. TadA dual base editor (TadDE)‐ like technologies that can catalyze both A to I and C to U editing and perform both the functions of ABE and CBE may be the first in this direction.^[^ ^120^ ^]^ Recent reports on deaminase‐free glycosylase base editors have suggested that the focus is also on minimizing the size of the effector domains.^[^ ^121^ ^]^ Temporal control of the editor's activities could improve safety.^[^ ^122^ ^]^ The field is also moving toward safer approaches that avoid DNA nicks. Emerging epigenome editing techniques can modify gene activity without the need for DNA alterations. Ongoing research into the mechanism of epigenome editing and its memory is expected to provide options for reversible and irreversible editing.^[^ ^123^ ^]^ Epigenome editing is currently being tested for hematological disorders, with studies showing that epigenome modifications are maintained in HSPCs post‐transplantation in small animal models. Integrase‐based technologies are promising for complementing these developments. Novel‐engineered recombinases and transposases have demonstrated encouraging efficiencies in cell lines. Additionally, recently reported Bridge RNAs, which utilize a DNA bridge recombinase mechanism, offer new possibilities for large genome modifications.

This review offers a comprehensive overview of recent advancements in prime editing, emphasizing its transformative potential in precision medicine while addressing challenges in current gene‐editing therapies such as efficiency, specificity, and safety and summarizing ongoing research and clinical trials aimed at bridging existing gaps and driving therapeutic innovation. As we continue to improve gene editing techniques, unlock new possibilities in the life sciences, paving the way for novel treatments and a deeper understanding of biological processes.

## Conflict of Interest

The authors declare no conflict of interest.
